# Comparative effectiveness of prophylactic antibiotic regimens in preventing infection in open fractures: a prospective cohort study

**DOI:** 10.1186/s12891-025-09063-3

**Published:** 2025-08-21

**Authors:** Mohammad Sheibani, Amirreza Sadeghifar, Shahriyar Shafa, Salman Azarsina, Mohammad Sajjad Mirhoseini, Mahdi Mohammaditabar, Elham al-Sadat Seyyed Ghasemi, Nima Rafati

**Affiliations:** 1 Clinical Research Development Unit, School of Medicine, Shahid Madani Hospital, Alborz University of Medical Sciences, Karaj, Iran; 2 Orthopedic Department, Afzalipour School of Medicine, Kerman University of Medical Sciences, Kerman, Iran; 3 Ophtalmology department, Kerman University of Medical Sciences, Kerman, Iran; 4 Student Research Committee, School of Medicine, Alborz University of Medical Sciences, Karaj, Iran; 5 Student Research Committee, School of Medicine, Iran University of Medical Sciences, Tehran, Iran; 6 Department of Orthopedics, School of Medicine, Clinical Research Development Unit, Shahid Madani Hospital, Alborz University of Medical Sciences, Karaj, Iran

**Keywords:** Open fractures, Prophylactic antibiotics, Vancomycin, Cefazolin, Infection prevention, Cohort study

## Abstract

**Background:**

Open fractures, particularly Gustilo-Anderson grade III injuries, carry high risks of infection. This cohort study compared the effectiveness of three prophylactic antibiotic regimens in reducing a range of infection-related outcomes, including infection markers (ESR, CRP), wound colonization, clinical infections, fever, cellulitis, and abscess formation.

**Methods:**

In this prospective cohort study, 600 patients aged 18–85 years with open fractures were enrolled at Shahid Bahonar Hospital, Iran (2020–2022). Participants were grouped by clinician-selected regimens: Group A (Cefazolin 1 g every 6 h + amikacin), Group B (Cefazolin 2 g every 8 h + amikacin), and Group C (Vancomycin 2 g every 12 h + amikacin). Multivariable logistic regression adjusted for confounders (age, fracture severity). Missing data (< 5%) were imputed.

**Results:**

Of 978 patients screened, 600 completed follow-ups. Baseline demographics (mean age: 31.2 years; 82% male) were balanced. Group C had lower rates of elevated ESR (4.8% vs. 8.0% in Group A; adjusted RR = 0.61, 95% CI: 0.40–0.92), clinical infection (4.7% vs. 8.0%; RR = 0.58, CI: 0.38–0.89), and deep infection (2.7% vs. 5.3%; RR = 0.51, CI: 0.29–0.90). Minor adverse events (rash/nausea) were balanced across all groups (5.5% overall), with no significant differences in incidence or type between regimens.

**Conclusion:**

Vancomycin-amikacin was associated with reduced infection markers and complications compared to cefazolin regimens in this observational cohort, supporting its consideration in high-risk settings. Causality requires confirmation through randomized trials.

**Supplementary Information:**

The online version contains supplementary material available at 10.1186/s12891-025-09063-3.

## Introduction

Open fractures represent a significant challenge in orthopedic trauma care due to their high risk of infection and the resulting complications such as nonunion, osteomyelitis, and even limb loss. By definition, an open fracture is characterized by a break in the skin that communicates with the fracture site, allowing environmental contaminants to invade the wound and predisposing it to bacterial colonization [1]. The Gustilo–Anderson classification remains the most widely used system to stratify these injuries according to wound size, degree of contamination, and soft-tissue damage [2]. This classification not only guides surgical management but also informs the choice and duration of prophylactic antibiotic therapy.

Historically, prophylaxis in open fractures has relied on first-generation cephalosporins (e.g., cefazolin) because of their efficacy against gram-positive cocci and, when combined with an aminoglycoside, a moderate spectrum of gram-negative organisms [3, 4]. However, increasing rates of multidrug-resistant bacteria, including methicillin-resistant *Staphylococcus aureus* (MRSA), have prompted a reassessment of standard regimens. Recent studies suggest that the addition of vancomycin—either alone or in combination with other agents—may better cover resistant organisms and thereby reduce surgical site infections (SSIs) in high-grade injuries [5].

In addition to the choice of antibiotic, the timing of administration is critical. Current evidence supports the administration of prophylactic antibiotics within the first hour of injury, as delays are associated with higher infection rates [5]. Despite these recommendations, controversies remain regarding the optimal dosing regimen and duration of therapy, especially for Gustilo–Anderson type III fractures that involve extensive soft tissue injury and contamination [6].

Our study was designed as a prospective cohort study to compare three prophylactic regimens in patients with severe (Gustilo–Anderson type III) open fractures. Two groups received cefazolin—with different dosing intervals—in combination with amikacin, while the third group was treated with a regimen of vancomycin plus amikacin. We evaluated both clinical outcomes (such as colonization, clinical infection, fever, cellulitis, deep infection, and abscess formation) and laboratory parameters (ESR and CRP levels) over a six-month follow-up period. Through this investigation, we aim to contribute to the evolving literature on antibiotic prophylaxis by clarifying whether broader-spectrum coverage—including agents effective against MRSA—can provide superior protection in this high-risk patient population.

## Materials and methods

### Study design and setting

This prospective cohort study was conducted at Shahid Bahonar Hospital, Kerman University of Medical Sciences, in Kerman, Iran, between January 2020 and December 2022. The study protocol was approved by the ethics committee of Kerman University of Medical Sciences IR.kmu.REC.1394.528, and informed consent was obtained from all participants.

#### Participants

Consecutive eligible patients presenting with open fractures or impending open fractures were enrolled. Three exposure groups were formed based on the prophylactic antibiotic regimen administered during standard care:

- Group A: Cefazolin 1 g IV every 6 h plus amikacin.
- Group B: Cefazolin 2 g IV every 8 h plus amikacin.
- Group C: Vancomycin 2 g IV every 12 h plus amikacin.

##### Inclusion criteria

- Age 18–85 years.
- Open fracture or closed fracture at risk for skin necrosis (impending open fracture, defined as fractures with significant soft tissue compromise, such as severe swelling, ecchymosis, or tenting of the skin, indicating a high likelihood of progression to an open fracture).
- Ability to provide informed consent and comply with follow-up.

##### Exclusion criteria

- Age < 18 or > 85 years.
- Fractures requiring multiple surgeries or polytrauma needing additional interventions.
- Hypersensitivity to vancomycin, cefazolin, or aminoglycosides.
- Prior MRSA infection, recent limb surgery (< 1 year), or antibiotic use within two weeks of injury.
- Inability to comprehend study requirements.

#### Exposure assessment

Antibiotic regimens were selected by treating clinicians based on hospital protocols and clinical judgment. All patients underwent uniform initial debridement and wound irrigation (without pulsed irrigation). Tetanus prophylaxis was administered per WHO guidelines. The vancomycin regimen of 2 g every 12 h was selected to ensure adequate tissue penetration and coverage against potential MRSA infections, given the high-risk nature of open fractures. Amikacin was administered at a dose of 15 mg/kg once daily for 3 days in all groups. The duration of antibiotic administration was 3 days for all regimens.

#### Outcome measures

##### Primary outcomes

1. Incidence of elevated inflammatory markers (ESR > 30 mm/hr, CRP > 10 mg/L).
2. Wound contamination/colonization, defined as any bacterial growth on culture taken during initial debridement, without quantitative thresholds, as per standard microbiological reporting at our institution.
3. Clinical infection (purulent drainage, erythema, systemic signs).
4. Deep infection (bone or deep tissue involvement).

##### Secondary outcomes

1. Fever (> 38 °C for > 24 h).
2. Cellulitis, abscess formation.
3. Need for reoperation or prolonged hospitalization.

#### Follow-up and data collection

Participants were monitored daily during hospitalization. Laboratory tests (ESR, CRP, WBC) were performed on admission and at follow-up visit at 6 months post-discharge. Wound cultures were obtained during initial debridement. Trained staff collected data using standardized forms. Renal function was monitored daily during the administration of vancomycin and amikacin. Serum creatinine levels were checked, and doses were adjusted if there was any sign of renal impairment. At the 6-month follow-up, patients were assessed for signs of delayed infection, nonunion, or other complications related to the fracture.

#### Sample size justification

A target sample size of 600 patients (200 per group) was chosen to detect a 10% difference in infection rates (α = 0.05, power = 80%), accounting for 10% attrition.

### Statistical analysis

Data were analyzed using SPSS version 14. Analyses followed intention-to-treat principles. Continuous variables (mean ± SD) were compared using independent t-tests; categorical variables were analyzed with chi-square or Fisher’s exact tests. Missing data (< 5% across groups) were addressed using multiple imputation with chained equations, generating five imputed datasets, and results were pooled using Rubin’s rules. A two-sided p-value < 0.05 was considered statistically significant. No adjustments for multiple comparisons were applied to primary outcomes.

## Results

### Participant flow and follow-up

Between January 2020 and December 2022, 1002 patients with open or impending open fractures were screened. After excluding 402 patients (142 did not meet inclusion criteria, 85 declined participation, 161 met exclusion criteria, 24 did not attend for the follow-up visit), 600 completed the study and were analyzed in three exposure groups based on clinician-selected antibiotic regimens: Group A (Cefazolin 1 g + amikacin, *n* = 200), Group B (Cefazolin 2 g + amikacin, *n* = 200), and Group C (Vancomycin + amikacin, *n* = 200).

### Baseline characteristics

Demographic and clinical characteristics were comparable across groups (Table 1). The cohort had a mean age of 31.24 ± 9.69 years, with no significant differences in age, gender distribution (82% male, 18% female), fracture type (open vs. impending open), or Gustilo-Anderson grades (*p* > 0.05 for all).

**Table 1:** Baseline demographics by treatment group

**Parameter**	**Group A (Cefazolin 1g)**	**Group B (Cefazolin 2g)**	**Group C (Vancomycin)**	*p*-value
N	200	200	200	–
Mean age (years)	30.63 ± 9.40	31.07 ± 10.11	32.03 ± 9.55	0.195
Gender (Male/Female)	159/41	164/36	169/31	0.429

### Infection and inflammatory outcomes

After adjusting for age, Group C showed significantly lower rates across multiple outcomes compared to Group A: elevated ESR (adjusted RR = 0.61, 95% CI: 0.40–0.92), elevated CRP (adjusted RR = 0.50, 95% CI: 0.33–0.75), wound colonization (adjusted RR = 0.67, 95% CI: 0.47–0.95), clinical infection (adjusted RR = 0.58, 95% CI: 0.38–0.89), fever (adjusted RR = 0.58, 95% CI: 0.38–0.89), cellulitis (adjusted RR = 0.58, 95% CI: 0.38–0.89), deep infection (adjusted RR = 0.51, 95% CI: 0.29–0.90), and abscess formation (adjusted RR = 0.37, 95% CI: 0.17–0.81). Detailed results are presented in Table 2.

**Table 2:** Summary of laboratory and clinical outcomes

**Outcome**	**Group A (%)**	**Group B (%)**	**Group C (%)**	**Adjusted RR (Group C vs. A)**	**95% CI**	*p*-value
Positive ESR	8.0	7.5	4.8	0.61	0.40–0.92	0.019
Positive CRP	9.3	8.3	4.7	0.50	0.33–0.75	0.001
Colonization	10.0	10.3	6.7	0.67	0.47–0.95	0.023
Clinical Infection	8.0	7.5	4.7	0.58	0.38–0.89	0.012
Fever	8.0	7.5	4.7	0.58	0.38–0.89	0.012
Cellulitis	8.0	7.5	4.7	0.58	0.38–0.89	0.012
Deep Infection	5.3	4.8	2.7	0.51	0.29–0.90	0.021
Abscess Formation	3.7	2.8	1.3	0.37	0.17–0.81	0.013

Adjusted RRs and CIs are calculated using multivariable Poisson regression with robust standard errors, adjusted for age

## Discussion

This prospective cohort study evaluated the comparative effectiveness of three prophylactic antibiotic regimens in preventing infection among patients with open fractures. The findings suggest that the vancomycin-based regimen (Group C) was associated with significantly lower rates of infection markers and clinical outcomes compared to cefazolin-based regimens (Groups A and B). These differences persisted after adjusting for confounders such as age, highlighting the potential role of broader-spectrum regimens in high-risk settings. These results align with existing evidence supporting the role of vancomycin in mitigating infections in open fractures, particularly in settings where resistant organisms like MRSA are prevalent [5, 7]. Below, we contextualize these findings within the broader literature, address methodological considerations, and outline implications for clinical practice.

The vancomycin plus amikacin regimen (Group C) was associated with lower rates of ESR, CRP, colonization, and clinical infection (Table 2), potentially due to its broader Gram-positive coverage, including MRSA, compared to cefazolin [5, 7]. The 4.7% clinical infection rate in Group C contrasts with 7.5–8.0% in cefazolin groups, suggesting a possible advantage in high-risk injuries [5]. This is consistent with Mario et al. [8], who noted reduced infection rates with vancomycin-containing regimens in grade III fractures. The addition of amikacin, targeting Gram-negative organisms [9], may also contribute to the lower deep infection and abscess rates in Group C (2.7% and 1.3%, respectively).

Interestingly, no significant differences were observed between the two cefazolin dosing regimens (Groups A and B), indicating that increasing the dose or altering the interval may not enhance efficacy in this context. This suggests a potential ceiling effect for cefazolin, where its spectrum of activity limits additional benefits beyond a certain threshold, regardless of dosing adjustments. This finding aligns with Mario et al. [8], who reported no difference between cefazolin alone and vancomycin-cefazolin combinations in some settings, but contrasts with studies favoring cefazolin for lower-grade fractures [9].

This study adhered to STROBE guidelines, employing rigorous outcome assessment, multivariable adjustment, and a robust sample size (*n* = 600) to address observational biases. However, limitations include the single-center design at Shahid Bahonar Hospital, which may limit generalizability to regions with differing MRSA prevalence or antibiotic stewardship practices. Additionally, unmeasured confounders (e.g., variations in surgical technique or postoperative care) could influence outcomes. The observational nature of the cohort design precludes causal conclusions, as antibiotic selection was based on clinician judgment rather than randomization. While our findings suggest that the vancomycin-amikacin regimen is associated with lower infection rates, further randomized controlled trials are needed to confirm these associations. Second, while all patients received systemic antibiotics, the protocol did not incorporate local antibiotic delivery, which meta-analyses suggest could further reduce infection rates by 4.6–16.5% when combined with systemic prophylaxis [8]. Third, follow-up was limited to six months, potentially underestimating delayed infections. Finally, the exclusion of polytrauma patients and those requiring multiple surgeries limits applicability to more complex cases.

These findings support reconsidering prophylactic regimens in open fractures, particularly in settings with high MRSA prevalence. Vancomycin-amikacin combinations may be preferable for severe injuries (Gustilo-Anderson grade II/III), as supported by EAST guidelines [5]. However, antibiotic stewardship remains critical [10] demonstrated that protocol-driven reductions in aminoglycoside use did not increase infection rates, underscoring the need for balanced approaches to avoid resistance.

The study also highlights the potential for adjunctive local antibiotic therapies. While systemic prophylaxis remains foundational, local aminoglycoside administration or vancomycin powder could further reduce infections, as evidenced by multiple studies [8, 11]. Future protocols should explore hybrid strategies combining targeted systemic agents with localized delivery.

## Conclusion

In this cohort study, vancomycin-amikacin prophylaxis was associated with reduced infection-related complications compared to cefazolin-based regimens in patients with open fractures. These findings align with guidelines emphasizing tailored antibiotic selection in settings with high MRSA prevalence. While broader-spectrum regimens may benefit high-risk populations, their use must balance efficacy against antibiotic resistance risks. Future multi-center studies with standardized protocols and longer follow-up are needed to validate these associations and refine prophylactic strategies.

## Supplementary Information

Supplementary Material 1.

## Data Availability

All data are available upon request from the corresponding author (Email: Nimarafati2273@gmail.com).

## Declarations

### STROBE compliance

Prospective design, exposure classification, confounder adjustment, outcome blinding, and attrition reporting.
