# Supplementary material for: Comparative effectiveness of prophylactic antibiotic regimens in preventing infection in open fractures: a prospective cohort study
Source: BMC Musculoskelet Disord. 2025 Aug 21;26:809. doi: 10.1186/s12891-025-09063-3 (PMC12369074; doi:10.1186/s12891-025-09063-3)
Supplement: Supplementary file 1 — Supplementary Material 1. [file 12891_2025_9063_MOESM1_ESM.docx]

**Table 1. Baseline Demographics by Treatment Group**

| **Parameter** | **Group A (Cefazolin 1g)** | **Group B (Cefazolin 2g)** | **Group C (Vancomycin)** | **p-value** |
| --- | --- | --- | --- | --- |
| N | 200 | 200 | 200 | – |
| Mean Age (years) | 30.63 ± 9.40 | 31.07 ± 10.11 | 32.03 ± 9.55 | 0.195 |
| Gender (Male/Female) | 159/41 | 164/36 | 169/31 | 0.429 |

**Table 2. Summary of Laboratory and Clinical Outcomes**

| **Outcome** | **Group A (%)** | **Group B (%)** | **Group C (%)** | **Adjusted RR (Group C vs. A)** | **95% CI** | **p-value** |
| --- | --- | --- | --- | --- | --- | --- |
| Positive ESR | 8.0 | 7.5 | 4.8 | 0.61 | 0.40–0.92 | 0.019 |
| Positive CRP | 9.3 | 8.3 | 4.7 | 0.50 | 0.33–0.75 | 0.001 |
| Colonization | 10.0 | 10.3 | 6.7 | 0.67 | 0.47–0.95 | 0.023 |
| Clinical Infection | 8.0 | 7.5 | 4.7 | 0.58 | 0.38–0.89 | 0.012 |
| Fever | 8.0 | 7.5 | 4.7 | 0.58 | 0.38–0.89 | 0.012 |
| Cellulitis | 8.0 | 7.5 | 4.7 | 0.58 | 0.38–0.89 | 0.012 |
| Deep Infection | 5.3 | 4.8 | 2.7 | 0.51 | 0.29–0.90 | 0.021 |
| Abscess Formation | 3.7 | 2.8 | 1.3 | 0.37 | 0.17–0.81 | 0.013 |
| *Note:* Adjusted RRs and CIs are calculated using multivariable Poisson regression with robust standard errors, adjusted for age. | | | | | | |
